# Oral cryotherapy by Kangfuxin for prophylaxis of oral mucositis in patients undergoing autologous hematopoietic stem cell transplantation

**DOI:** 10.1097/MD.0000000000042867

**Published:** 2025-07-04

**Authors:** Dan Chen, Fang-fang Li, Hong-mei Zhu, Xiao Wang, Xiao-mei Zhang, Ting Ma, Hui Zhang, Yan-qiong Ma, Xin-miao Wang, Yong-hua Wang, Hao Yao, Hai Yi

**Affiliations:** a Department of Hematology, People’s Liberation Army The General Hospital of Western Theater Command, Chengdu, China; b Department of Nursing, People’s Liberation Army The General Hospital of Western Theater Command, Chengdu, China.

**Keywords:** autologous hematopoietic stem cell transplantation, Kangfuxin solution, oral cryotherapy, oral mucositis

## Abstract

Oral mucositis is a common complication in patients undergoing autologous hematopoietic stem cell transplantation (auto-HSCT), and currently, there is no universally effective treatment. This study aims to evaluate the preventive and delaying effects of oral cryotherapy using Kangfuxin (KFX) on oral mucositis in auto-HSCT patients. A total of 86 patients, treated between January 2020 and August 2023, were randomly assigned into 2 groups: the control group (40 cases) received cryotherapy using frozen physiological saline ice cubes, and the experimental group (46 cases) received KFX ice cubes. Both treatments started 30 minutes before melphalan infusion, and continued until the end of the infusion. The primary outcome was the incidence and severity of oral mucositis, with secondary outcomes including pain levels and patient-reported general condition. The experimental group demonstrated a significantly higher proportion of patients with grade 0 oral mucositis compared to the control group. Furthermore, the incidence of grade III–IV oral mucositis in the control group was 32.5%, compared to 10.8% in the experimental group, showing a statistically significant difference (*P* < .05). The experimental group had fewer days of using lidocaine mouthwash and lower pain scores, showing statistically significant differences (*P* < .05). In conclusion, our findings suggest that KFX oral cryotherapy can be an effective approach for preventing and reducing the severity of oral mucositis in patients undergoing auto-HSCT. It may help alleviates the symptoms such as pain and discomfort, thereby improving patient well-being during treatment.

## 1. Introduction

Hematopoietic stem cell transplantation is an effective treatment for hematological malignancies such as lymphoma, multiple myeloma, and myelodysplastic syndrome.^[1,2]^ Patients receive high-dose chemotherapy as a conditioning regimen, which has a cytotoxic effect on normal cells, especially rapidly proliferating oral mucosal cells, leading to toxic inflammatory reactions in the epithelial cells of the oral mucosa.^[3]^ As a result, hematopoietic stem cell transplantation patients are prone to developing oral mucositis. Studies found that the incidence of oral mucositis in auto-HSCT patients is approximately 35.0% to 75.0%.^[3,4]^

The clinical manifestations of oral mucositis are as follows: Local redness of the oral mucosa is the initial presentation, followed by white patches, tenderness, and in severe stages, epidermal detachment, scab formation, and cellulose exudation leading to the formation of pseudomembranes and ulcers.^[5]^ Oral mucositis can cause unbearable pain, broad ulceration, double the risk of infection, prolonged hospitalization, increased hospitalization costs, and other adverse factors.^[5,6]^ Relevant studies have indicated that prophylactic medication and appropriate oral care can prevent or alleviate the occurrence of oral mucositis in patients, accelerate its healing, and improve patients’ quality of life.^[5–7]^ During cytotoxic chemotherapy, particularly with agents such as melphalan and 5-fluorouracil, continuous mouth rinsing with ice cubes or ice chips can cause vasoconstriction of the oral mucosal blood vessels, reduce the distribution of cytotoxic drugs in the oral mucosa, and effectively lower the incidence of oral mucositis.^[7,8]^ However, the cryogenic medium used in clinical practice is mostly pure water or sterile saline, which provides a cooling effect but no additional protective or regenerative properties for the oral mucosa. Based on clinical practice, our department adopted Kangfuxin (KFX) as the cryogenic medium for cryotherapy in patients undergoing auto-HSCT, achieving significant preventive effects.

KFX liquid is a traditional Chinese medicine derived from the dried whole body of American cockroaches (Periplaneta americana),^[9–11]^ which contains bioactive substances such as amino acids and peptides, which aid tissue repair, and improve vascular function.^[9,10,12]^ Cold condition, as used in oral cryotherapy, induce vasoconstriction, reduce oral blood flow, lower tissue temperature, and inhibit inflammatory spread. KFX may enhance the healing of oral ulcers by promoting granulation tissue formation, improving microcirculation, reducing redness, swelling, and pain, and increasing blood supply to damaged tissues, thereby creating a favorable environment for mucosal regeneration.^[9,10,12]^

## 2. Materials and methods

### 2.1. Ethical approval

Ethical approval was obtained from the Ethics Committee of People’s Liberation Army General Hospital of Western Theater Command (Approval Number: 2022ky060-1). Written informed consent was obtained from all participants prior to their enrollment in the study. Participants’ identities were anonymized by assigning unique codes during data collection, and the confidentiality of all data was maintained throughout the study. All procedures were conducted in accordance with the Declaration of Helsinki and relevant guidelines and regulations.

The use of KFX solution in this study was part of standard clinical care for managing oral mucositis and does not represent an experimental intervention.

Clinical trial number: Not applicable, as this study was an observational study and not a registered clinical trial.

### 2.2. Participants and randomization

A total of 86 patients who underwent auto-HSCT at our center from January 2020 to August 2023 were included in this study. All eligible patients were enrolled, with no exclusions during the recruitment phase. Patients were randomly assigned to either the control group (n = 40) or the experimental group (n = 46) using a computer-generated randomization sequence. The unequal group sizes resulted from this randomization process. All 86 randomized patients received their allocated intervention, completed the study protocol, and were included in the final analysis. There were no dropouts or losses to follow-up in either group. This was an open-label study.

#### 2.2.1. Inclusion and exclusion criteria

Patients aged 18 to 70 years, with a confirmed diagnosis of hematological malignancies and who were eligible for auto-HSCT, were included in the study. Patients were required to have adequate organ function and no active infection. Additionally, patients who developed oral mucositis (prior to the intervention) during previous chemotherapy were also included, as this population is at a higher risk of developing oral mucositis again.

Patients were excluded if they had a history of severe oral disease unrelated to mucositis, were actively infected at the time of the study, or had any condition contraindicating the use of cryotherapy. Patients with an inability to comply with the study protocol were also excluded.

#### 2.2.2. Sample size determination and randomization

This was a single-center, open-label, randomized pilot study. Participants were randomly assigned to either the control or experimental group using a computer-generated randomization sequence. As an open-label trial, both participants and investigators were aware of the treatment assignments. Given the exploratory nature of the study, the sample size was based on feasibility, and no formal sample size calculation was performed.

### 2.3. Conditioning regimen and cryotherapy intervention

#### 2.3.1. Transplantation preconditioning regimen

Patients underwent various conditioning regimens prior to auto-HSCT. The common feature among all regimens was the inclusion of melphalan. The conditioning protocols included, but were not limited to:

(1) BEAM: Carmustine (BCNU), Etoposide, Cytarabine (Ara-C), and Melphalan;
(2) VPM: Etoposide (VP16), Cytarabine (Ara-C), and Melphalan;
(3) BEAC: Bendamustine, Etoposide, Cytarabine (Ara-C), and Melphalan.

The specific dosing and timing of each agent varied based on the individual protocol. However, melphalan was consistently administered on day-1 of the transplantation process.

Detailed information about each patient’s conditioning regimen, including specific agents, doses, and administration schedules, has been compiled and is provided in the supplementary materials.

This approach allows for standardized cryotherapy intervention across all patients, focusing on the administration of melphalan, which is common to all regimens and known to be associated with oral mucositis.

#### 2.3.2. Cryotherapy intervention

The patients in the study were divided into 2 groups. Both the control group and the experimental group received oral cryotherapy starting 30 minutes before melphalan infusion and continuing throughout the infusion, for a total duration of approximately 2.5 hours. This duration was chosen as it covers the critical period for preventing oral mucositis and represents the upper limit of what patients can comfortably tolerate without compromising compliance. The control group used frozen sterile saline ice cubes, while the experimental group used frozen KFX ice cubes. The size of the ice cubes used in both groups was 1.5 cm × 1.5 cm × l cm. Patients were instructed to continuously hold the ice cubes in their mouth, allowing them to melt gradually, and replacing them as needed to ensure constant cryotherapy. On average, patients used approximately 10 to 15 ice cubes/h, though individual consumption varied.

Melphalan was administered as a 2-hour intravenous infusion. The cryotherapy continued for 30 minutes after the completion of the melphalan infusion to cover the immediate post-administration period, which is crucial for mucositis prevention.

During the cryotherapy period, patients were advised to avoid consuming extremely hot or cold foods. When pain was occurred, 100 mL of 0.9% normal saline solution and 5 mL of lidocaine were used for rinsing. Patients were instructed to rinse with 10 to 15 mL of lidocaine mouthwash, moving it around the cheeks for >15 seconds to ensure full contact with the oral mucosa before spitting it out.

### 2.4. Evaluation of outcomes

The primary outcome of this study was the incidence and severity of oral mucositis, as assessed daily using the National Cancer Institute’s Common Toxicity Criteria (Table 2) for Adverse Events version 5.0^[13]^ from day +1 to day +20 posttransplantation.

**Table 2:** NCI common toxicity criteria.

Grade	Symptoms
0	No mucositis
1	Painless ulcers, erythema or mild soreness without lesions
2	Painful erythema, edema or ulcers but can swallow
3	Painful erythema, edema or ulcers preventing swallowing or requiring hydration or TPN
4	Severe ulceration requiring prophylactic intubation

NCI = National Cancer Institute, TPN = total parenteral nutrition.

The secondary outcomes included: Duration of oral mucositis. Pain levels, assessed using a 0 to 10 numerical rating scale. The number of days enteral nutrition was required.

Overall patient well-being, including aspects such as swallowing, eating, drinking, sleeping, and taste, based on a patient questionnaire.

Mucositis was evaluated daily by trained healthcare professionals who were not blinded to the treatment groups. The CTCAE scale was used to grade the severity of oral mucositis..

### 2.5. Statistical methods

Statistical analysis was performed using BM SPSS Statistics version 26.0 (IBM Coporation). The sample size was determined based on an expected reduction in the incidence of grade III–IV oral mucositis by 20%, with 80% power and a 2-sided significance level of 0.05. The chi-square test was used to compare categorical variables such as the incidence of grade III–IV mucositis, while continuous variables, including pain scores and days of lidocaine use, were analyzed using the *T*-test. Multivariable logistic regression analysis was performed to adjust for potential confounders, including baseline patient characteristics such as age, CD34 count, and chemotherapy regimen.

## 3. Results

### 3.1. General condition of patients

A total of 86 patients who underwent auto-HSCT were randomly divided into a control group (n = 40) and an experimental group (n = 46). The baseline characteristics of the 2 groups, including gender, age, and CD34-positive cell count, were compared, and no significant differences were found (*P* > .05), indicating comparability between the groups. Details are shown in Table 1.

**Table 1:** Daily patient-reported mouth pain scores, median of each group.

	Frozen physiological saline, n = 40	Frozen KFX solution, n = 46
Age, median (range)	51 (22–68)	24.5 (22–59)
Gender		
Male (number)	22	30
Female (number)	18	16
CD34+ cells/kg, median (range)	2.6 (1.31–15.38)	3.1 (0.73–20.2)
Day absolute neutrophil count 4 > 500, median (range)	10.0 (7–14)	10.0 (6–15)
Units platelets transfused, median (range)	2.4 (0.8–13)	3.0 (0.5–9.8)
Units RBCs transfused (%)	10 (4%)	6 (13%)

KFX = Kangfuxin, RBCs = red blood cells.

### 3.2. OM grading

Oral mucositis was graded daily using the National Cancer Institute’s OM grading standards, based on ulcer symptoms. his allowed for timely detection and appropriate treatment interventions. Details are shown in Table 2 ^[14]^ for grading criteria.

### 3.3. Mucositis grading and associated patient outcomes

Following the intervention, the incidence of oral mucositis in the control group was as follows: grade I: 4 cases, grade II: 8 cases, grade III: 8 cases, grade IV: 5 cases, with grade III–IV accounting for 32.5%. In the experimental group, the incidence was grade I: 8 cases, grade II: 12 cases, grade III: 4 cases, and grade IV: 1 case, with grade III–IV accounting for 10.8%. The difference between the 2 groups was statistically significant (*P* < .05). The median duration of enteral nutrition was 10 days in the control group and 6.5 days in the experimental group, with no statistically significant difference (*P* > .05). However, the median length of hospital stay was 24.5 days in the control group and 27 days in the experimental group, with a statistically significant difference (*P* < .05), though other factors may have contributed to this difference. Furthermore, the median duration of oral ulcer pain in the experimental group was 2 days, significantly lower than in the control group, with a statistically significant difference (*P* < .05). Details are shown in Table 3.

**Table 3:** Results of mucositis scores and secondary events.

	Frozen physiological saline, n = 40	Frozen KFX solution, n = 46	*P*
Maximum NCI mucositis grade			
Grade 0	15	21	
Grade 1	4	8	
Grade 2	8	12	
Grade 3	8	4	
Grade 4	5	1	
Incidence of grade 3–4 mucositis	32.5%	10.8%	.013
Days of TPNa*	10 (0–20)	6.5 (0–26)	.624
Days of hospitalization*	24.5 (17–53)	27 (18–48)	.045
Lidocaine mouthwash, d*	3 (1–5)	2 (1–3)	.036
Weight loss, %*^,^†	1.9 (0.1–9.5)	2.5 (0.2–8.3)	

KFX = Kangfuxin, NCI = National Cancer Institute, TPN = total parenteral nutrition.

* Median (range).

† At day 21.

### 3.4. Patient-reported outcomes

The outcomes related to swallowing, eating, drinking, speaking, sleeping, and taste, were assessed on days 1, 6, 11, 16, and 21 after transplantation for both groups. Significant differences were observed between the groups across all time points (*P* < .05), suggesting a potential benefit of frozen KFX gargles in improving patient-reported oral function and comfort. Details are shown in Table 4.

**Table 4:** Patient-reported events.

	Frozen physiological saline, n = 40	Frozen KFX solution, n = 46	*P*
Pain	2.7	1.8	.029
Swallow	2.2	1.6	.027
Drink	1.7	1.3	.039
Eat	2.8	2.3	.035
Talk	1.4	1.1	.036
Sleep	1.5	1.8	.167
Taste	1.8	1.3	.012

Mean of the highest value for each patient.

KFX = Kangfuxin.

## 4. Discussion

Chemotherapy drugs are known to cause side effects such as decreased appetite and anorexia,^[15]^ and reduced saliva secretion, leading to an increased concentration of saliva, and decreased self-cleaning ability of oral mucosa.^[16]^ At the same time, chemotherapy drugs can also have inhibitory and killing effects on oral mucosa, directly affecting the regeneration, maturation, and repair of mucosal cells.^[15]^ These effects leads to dry oral mucosa, rapid bacterial growth, and fermentation of food residues^[1,15,16]^ ultimately resulting in ulceration and erosion of the mucosa.^[17]^ Acidic substances in the oral cavity further exacerbate ulceration, prolonging wound healing and affecting diet and creating a vicious cycle.^[18–20]^

Prior to this study, KFX was known for its tissue repair and anti-inflammatory properties in traditional Chinese medicine. However, its application in oral mucositis prevention and treatment, particularly in the form of cryotherapy, had not been explored. In light of these challenges, this study investigates the use of frozen KFX cryotherapy as a novel approach to prevent and alleviate oral mucositis in patients undergoing chemotherapy. By targeting the inflammatory and ulcerative processes caused by chemotherapy, frozen KFX cryotherapy has the potential to break this cycle, promoting faster healing and improving patient outcomes.

Currently, physiological saline mouthwash is the commonly used treatment method for oral mucositis in clinical practice.^[21]^ Saline mouthwash helps reduce food residue, prevents bacterial growth, and maintains a normal oral environment, alleviating dryness due to reduced saliva secretion reduced saliva secretion.^[6,7,21]^ However, it lacks antibacterial properties and shows limited therapeutic effects on oral ulcers.^[16,21]^ Lidocaine mouthwash is often used to treat oral pain, which can achieve the effect of local analgesia.^[22]^ Lidocaine is an amide local anesthetic with strong penetration, good diffusion, rapid onset, long duration of action, and good analgesic effect.^[22]^ However, lidocaine does not promote mucosal healing and has limited therapeutic effects on oral ulcers, presenting significant disadvantages.^[20–22]^ Based on clinical experience, we implemented frozen KFX gargles in patient undergoing auto-HSCT, yielding promising effect.

The results suggest that frozen KFX gargles significantly prevented and delayed oral mucositis onset, relieved oral pain, and improved patients’ quality of life. Thus, frozen KFX mouthwash may be an economical, safe, and potentially effective supportive therapy for patients undergoing auto-HSCT. This study contributes new knowledge by demonstrating the efficacy of frozen KFX cryotherapy in preventing and alleviating oral mucositis in patients undergoing auto-HSCT, a novel application that combines the benefits of cryotherapy with the healing properties of KFX.

While our results are promising, this study has several limitations. In addition to the small sample size, which limits generalizability, the study was not double-blind, potentially introducing bias. Assessor training, while based on standardized protocols, may have varied, affecting the consistency of evaluations. Furthermore, the use of modified assessment tools adapted from previous studies may impact the comparability of our results with other research. These limitations highlight the need for future larger, multicenter, double-blind studies with standardized assessment protocols to more definitively assess the clinical efficacy of frozen KFX cryotherapy in managing oral mucositis.

## Author contributions

**Conceptualization:** Dan Chen.

**Data curation:** Xiao Wang, Hui Zhang, Yan-qiong Ma.

**Formal analysis:** Hong-mei Zhu, Yan-qiong Ma.

**Funding acquisition:** Dan Chen, Hao Yao.

**Investigation:** Fang-fang Li, Ting Ma, Hao Yao.

**Methodology:** Fang-fang Li, Xiao Wang, Xin-miao Wang.

**Project administration:** Fang-fang Li, Hong-mei Zhu, Xiao-mei Zhang, Ting Ma.

**Resources:** Xiao-mei Zhang.

**Supervision:** Dan Chen, Xin-miao Wang, Yong-hua Wang, Hao Yao, Hai Yi.

**Validation:** Hui Zhang.

**Writing – review & editing:** Hao Yao.
